# Complications of Treating Terrible Triad Injury of the Elbow: A Systematic Review

**DOI:** 10.1371/journal.pone.0097476

**Published:** 2014-05-15

**Authors:** Hong-wei Chen, Guo-dong Liu, Li-jun Wu

**Affiliations:** 1 Department of Orthopedics, Wenzhou Medical College-Affiliated Yiwu Central Hospital, Yiwu, Zhejiang, China; 2 Department 8, Research Institute of Surgery, Daping Hospital, Third Military Medical University, Chongqing, P.R. China; 3 Department of Orthopedics, Wenzhou Medical College-Affiliated Second Hospital, Wenzhou, Zhejiang, China; Institute of Digital Medicine, Wenzhou Medical College, Wenzhou, Zhejiang, China; Bascom Palmer Eye Institute, University of Miami School of Medicine, United States of America

## Abstract

**Objective:**

Terrible triad injury of the elbow (TTIE), comprising elbow dislocation with radial head and coronoid process fracture, is notoriously challenging to treat and has typically been associated with complications and poor outcomes. The objective of this systematic review was to summarize the most recent available evidence regarding functional outcomes and complications following surgical management of TTIE.

**Methods:**

Medline, EMBASE, Cochrane Library, and Google Scholar were searched to identify relevant studies, which were included if they were retrospective or prospective in design, involved participants who had TTIE, and were published in English. Outcomes of interest were functional outcomes and complications.

**Results:**

Sixteen studies, involving 312 patients, were included in the systematic review. Mean follow up after surgery was typically 25 to 30 months. Mean Mayo elbow performance scores ranged from 78 to 95. Mean Broberg-Morrey scores ranged from 76 to 90. Mean DASH scores ranged from 9 to 31. The proportion of patients who required reoperation due to complications ranged from 0 to 54.5% (overall  = 70/312 [22.4%]). Most of these complications were related to hardware fixation problems, joint stiffness, joint instability, and ulnar neuropathy. The most common complications that did not require reoperation were heterotopic ossification (39/312 [12.5%] patients) and arthrosis (35/312 [11.2%] patients).

**Conclusions:**

The results of this systematic review indicate that functional outcomes after surgery for TTIE are generally satisfactory and that complications are common. Further research is warranted to determine which surgical techniques optimize functional outcomes and reduce the risk of complications.

## Introduction

The combination of elbow dislocation with both radial head and coronoid process fracture is notoriously challenging to treat and, as such, has been termed “terrible triad” injury of the elbow (TTIE) [1]. This type of elbow injury is typically due to low or high energy falls onto an outstretched hand, which results in valgus and axial compression of the supinated forearm [2]. This leads to failure of the lateral collateral ligament complex (the medial collateral ligament may also fail), dislocation of the elbow, and consequent fracture of the radial head and coronoid process [2], [3]. As a result of these injuries, the elbow is left in an unstable state that invariably requires surgical intervention. Unfortunately, due to the complexity of injury, outcomes have traditionally been poor, with long-term complications including stiffness, pain, arthritis, and joint instability [4].

The aim of surgery in managing TTIE is the restoration of stability of the humeroulnar and humeroradial joints, thus facilitating early postoperative elbow motion to reduce the likelihood of long-term joint stiffness or disability [3], [5]. Clearly, to optimize the chances of success, such surgery must adequately account for all three injury components of the terrible triad [3]. Over the years various reports have described surgical management of these fractures, with there being differences in the surgical approach used, the means of fixation, and the type of implant used in cases requiring replacement arthroplasty of the radial head [2],[3]. To date, however, there is no consensus as to the optimal means of surgical management. In 2011, Rodriguez-Martin and colleagues [6] published the results of a systematic review summarizing injuring patterns, treatment, and outcomes (including complications) in patients with TTIE. On the basis of findings from five studies included in the review (all published before 2009), the authors made a number of recommendations regarding the management of TTIE. Since publication of Rodriguez-Martin's [6] article, the findings from a considerable number of additional studies on this topic have been published. Such studies clearly reflect the most current treatment practices. As a consequence, we decided to perform a systematic review of the literature to gain a more comprehensive understanding of complications and functional outcomes in patients with TTIE following surgical repair.

## Materials and Methods

### Search Strategy

Medline, EMBASE, Cochrane Library, and Google Scholar were searched on 31 July 2013 using combinations of the following search terms: elbow triad terrible, coronoid fracture, radial fracture, elbow fracture, and elbow dislocation.

### Study Selection

Studies were considered for inclusion in the review if they involved participants who met the criteria for TTIE (i.e., elbow dislocation, radial head fracture, and coronoid process fracture), and were published in English. Studies were excluded from the review if they included patients with injuries other than TTIE or if they were published in the form of letters, comments, editorials, or case reports. References lists of pertinent articles were hand searched to identify other potentially relevant studies.

### Data Extraction

Data were extracted by two independent reviewers who consulted with a third reviewer to resolve any disagreements. The following data/information were extracted from each eligible study: author details, number, sex, and age of patients, length of follow up, Mason classification for radial head fractures [7], Regan-Morrey classification for coronoid fractures [8], functional outcomes (Mayo elbow performance scores [1], Broberg-Morrey scores [9], and Disabilities of the Arm, Shoulder and Hand (DASH) scores [10]), and any complications.

### Outcome Measures

The outcomes measures of interest were functional outcomes and complications.

## Results

### Study Selection

**Figure 1:**
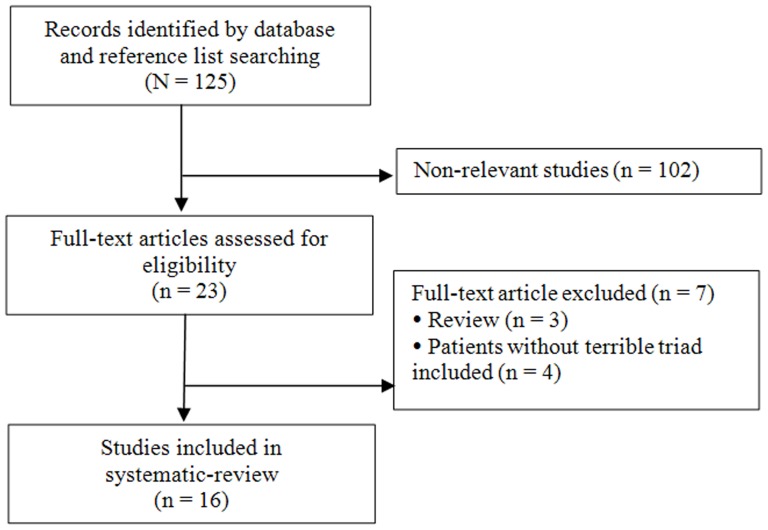
Flow diagram of study selection.

A total of 125 studies were identified in the initial search (Figure 1). Of these, 23 underwent full text review, 7 were excluded for various reasons (outlined in Figure 1), and 16 [11]–[26] were included in the systematic review.

### Study Characteristics

The main characteristics of the studies included in the systematic review are summarized in Table 1. All studies, except for that reported by Zeiders and Patel [15], were retrospective in design. The number of patients in the studies ranged from 6 to 40 (total  = 312). Most (13 of 16) studies reporting information on patient sex included a majority (>50%) of male patients. Most studies reported that the mean age of patients was 40 to 49 years. The mean length of follow up ranged from 13.6 to 64 months, although 25 to 30 months follow up was most common.

**Table 1:** Characteristics of studies included in the systematic review.

1^st^ Author (year)	Patients, number	Sex, % male	Mean Age (range), years	Mean Follow-up (range), months	Radial Head Fracture Classification^a^	Coronoid Fracture Classification^b^
Leigh (2012)	23	52.2	43.5 (19–67)	40.6 (16–73)	I–III	I–III
Toros (2012)	16	68.8	34 (24–50)	34.5 (14–110)	II–III	I–II
Garrigues (2011)	40	55	48 (22–76)	24 (18–53)	NA	I–III
Jeong (2010)	13	53.8	43.8 (15–76)	25 (18–41)	I–III	I–III^c^
Wang (2010)	8	75	39 (20–52)	NA	II–III	I–II
Chemama (2010)	13	84.6	NA	63 (15–128)	I–III	I–II
Seijas (2009)	18	44.4	45 (17–77)	13.6 (4–38)	II–III	I–II
Winter (2009)	13	69.2	40 (18–77)	25 (15–48)	Non-reparable fracture	No fracture or I
Pai (2009)	6	100	26–54	26.4 (12–36)	NA	I
Lindenhovius (2008)	18	66.7	47 (22–76)	29 (10–53)	II–III	II
Zeiders (2008)	32	NA	NA	36 (12–60)	NA	NA
Egol (2007)	37	40.5	Male: 49 (28–68)	27 (12–105)	I–III	I–III
			Female: 57 (32–79)			
Forthman (2007)	22	63.6	48 (24–75)	29 (12–53)	II–III	II
van Riet (2005)	6	66.7	42 (27–58)	64 (18–112)	NA	II–III
Pugh (2004)	36	61.1	41.4 (13–76)	34 (20–65)	I–IV	I–III
Ring (2002)	11	54.5	49 (17–67)	NA	II–III	II

NA, not available.

a Based on Mason Classification.

b Based on Regan-Morrey Classification.

c Based on O'Driscoll Classification.

### Functional Outcomes

8 of 16 studies included assessment of Mayo elbow performance scores, which ranged (mean) from 78 to 95 (Table 2). Two studies [21], [23] reported that mean scores excellent, whereas mean scores in the remainder of the studies were good. Overall, for the studies reporting individual patient results (N = 155 patients), 61 (39%) patients had excellent scores, 66 (43%) had good scores, 23 (15%) had fair scores, and 5 (3%) had poor scores. 7 of 16 studies included assessment of Broberg-Morrey Scores, which ranged (mean) from 76 to 90. Two studies [22], [25] reported that mean scores excellent, whereas mean scores in the remainder of the studies were good. Overall, for the studies reporting individual patient results (N = 98 patients), 27 (28%) patients had excellent scores, 39 (40%) had good scores, 24 (24%) had fair scores, and 8 (8%) had poor scores. 8 of 16 studies reported DASH scores, which ranged (mean) from 9 to 31.

**Table 2:** Summary of functional outcomes for studies included in the systematic review.

	Mayo Elbow Performance Scores		Broberg-Morrey Scores		DASH Scores
1^st^ author (year)	Mean (range)	Detail	Mean (range)	Detail	Mean (range)
Leigh (2012)	NA	NA	NA	NA	9.16 (0–18.3)^a^
					10.83 (6.7–37.9)^b^
Toros (2012)	92.8 (85–100)	9 excellent; 7 good	NA	NA	9.1 (0–32)
Garrigues (2011)	NA	NA	90 (64–100)	NA	16 (0–43)
Jeong (2010)	95 (85–100)	10 excellent; 3 good	NA	10 excellent; 3 good	NA
Wang (2010)	78 (55–95)	2 excellent; 3 good; 2 fair; 1 poor	76 (51–95)	1 excellent; 3 good; 3 fair; 1 poor	31 (0–72)
Chemama (2010)	87 (75–100)	4 excellent; 10 good	NA	NA	NA
Seijas (2009)	NA	NA	NA	NA	NA
Winter (2009)	NA	NA	86.5 (55–100)	NA	NA
Pai (2009)	NA	NA	NA	NA	NA
Lindenhovius (2008)	88 (65–100)	6 excellent; 8 good; 2 fair; 0 poor	90 (64–100)	5 excellent; 10 good; 3 fair; 0 poor	15 (0–43)
Zeiders (2008)	NA	NA	NA	NA	23 (19–28)
Egol (2007)	81 (45–100)	7 excellent; 12 good; 9 fair; 1 poor	77 (33–100)	3 excellent; 10 good; 12 fair; 4 poor	28 (0–72)
Forthman (2007)	89 (65–100)	7 excellent; 9 good; 1 fair; 0 poor	88 (53–100)	6 excellent, 11 good, 3 fair, 2 poor	13.3 (0–43)
van Riet (2005)	NA	1 excellent; 1 good; 2 fair; 2 poor	NA	NA	NA
Pugh (2004)	88 (45–100)	15 excellent; 13 good; 7 fair; 1 poor	NA	NA	NA
Ring (2002)	NA	NA	76 (34–98)	2 excellent; 2 good; 3 fair; 1 poor	NA

DASH, Disabilities of the Arm, Shoulder and Hand; NA, not available.

a DASH score for radial head repair group.

b DASH score for radial head replacement group.

### Complications

The complications reported in the studies included in the systematic review are summarized in Table 3. The proportion of patients who required reoperation ranged from 0 to 54.5%, with most studies reporting that approximately 30% of patients experienced the need for reoperation. No patients required reoperation in 3 studies [15], [16], [23]. Overall, 70 of 312 (22.4%) patients experienced complications requiring reoperation. Complications requiring reoperation were typically because of problems related to hardware/fixation, joint stiffness, joint instability, or ulnar neuropathy. There were few instances of wound infection. The following is a brief summary of the results from studies in which >1 patient experienced the same complication. Leigh et al. [24] reported that 2 of 23 (8.7%) patients experienced symptomatic nonunion of the radial head and neck or were unable to regain a functional range of motion despite >6 months of rehabilitation. Garrigues et al. [22] reported that 3 of 40 (7.5%) patients experienced limited flexion, residual instability, or had an oversized radial prosthesis. Seijas et al. [18] reported that 3 of 18 (16.7%) experienced dislocation and that 2 of 18 (11.1%) patients experienced Essex-Lopresti lesions. Winter et al. [17] reported that 2 of 13 (15.4%) patients experienced a lack of appropriate physiotherapy necessitating lateral arthrolysis. Lindehovius et al. [25] reported that 2 of 18 (11.1%) patients experienced joint stiffness or ulnar neuropathy. Forthman et al. [26] reported that 4 of 22 (18.2%) patients experienced ulnar neuropathy and that 3 of 22 (13.6%) patients experienced joint stiffness. Egol et al. [14] reported that 2 of 37 (5.4%) patients experienced joint stiffness. Pugh et al. [12] reported that 4 of 36 (11.1%) patients experienced limited range of motion and that 2 of 36 (5.6%) patients experienced radioulnar synostosis. Ring et al. [11] reported that 5 of 11 (45.4%) patients experienced redislocation.

**Table 3:** Summary of complications reported in studies included in the systematic review.

1st author (year)	Reoperations, number (%)	Number of Patients, Indication for Reoperation (Method of Reoperation)	Number of Patients, Complications Not Requiring Reoperation (Complication Management)
Leigh (2012)	6 (26.1)	2, symptomatic nonunion of repaired radial head and neck fracture (radial head replacement)	None
		1, migration of Kirschner wire for radial head fixation (Kirschner wire removal)	
		1, persistent joint subluxation due to no initial repair of LCL complex (LCL complex repair + downsize radial head implant)	
		1, deep infection (surgical washout + antibiotics)	
		2, unable to regain functional range of motion despite >6 months rehab (removal of metalware + circumferential capsular release)	
Toros (2012)	0 (0)	None	6, grade I arthrosis^a^
			4, ulnar neuropathy
			NA, heterotopic ossification
Garrigues (2011)	11 (27.5)	3, residual instability for 18 months (1 total elbow arthroplasty, 2 NA)	5, heterotopic ossification
		3, oversized radial prosthesis (NA)	4, nonunion
		5, capsulectomy (3 ulnar nerve transposition due to limited flexion prior to capsulectomy, 2 NA)	3, failed fixation
			3, malunion
Jeong (2010)	1 (7.7)	1, ulnar neuropathy (ulnar nerve release)	2, heterotopic ossification
Wang (2010)	2 (25.0)	1, broken Kirschner wire for fixation of humeroradial joint (Kirschner wire removal)	3, residual subluxation
		1, pain and extension deficit due to plate (olecranon fixation plate removed)	2, heterotopic ossification
Chemama (2010)	2 (15.4)	1, persistent instability due to disinserted MCL (ligament repair + external fixation)	1, osteoarthritis
		1, severe pain on the lateral column (Swanson metal radial head prosthesis removal; ulnocarpal impingement subsequently noted)	
Seijas (2009)	6 (33.3)	2, Essex-Lopresti lesion (Darrach's osteotomy)	4, heterotopic ossification
		1, arthritic degeneration <1 year (total elbow arthroplasty)	4, mechanical blocking of pronation and supination
		3, unnoticed dislocation (1 external fixator, 2 Kirschner wires)	2, transient ulnar nerve injury
Winter (2009)	6 (46.2)	1, subluxation to due to overstuffing of the implant (NA)	1, medial dislocation due to fall after surgery (simple closed reduction)
		1, radial head prosthesis disassembly due to overstuffing of the implant (NA)	1, heterotopic ossification
		1, deep infection (hardware removal)	
		2, lack of appropriate physiotherapy (lateral arthrolysis)	
		1, capitellar erosion (NA)	
Pai (2009)	0 (0)	None	1, radial neuropraxia
			1, mild stiffness
			1, mild osteoarthritis of radiocapitellar joint
Lindenhovius (2008)	5 (27.8)	2, stiffness (1 elbow release + ulnar nerve transposition, 1 elbow release + ulnar nerve release + excision of anterior and posterior heterotopic bone)	9, grade I arthrosis^a^
		2, ulnar neuropathy (ulnar nerve transposition)	3, grade II arthrosis^a^
		1, wound infection (surgical debridement + irrigation)	
		1, sustained distal humerus fracture (open reduction + internal fixation)	
Zeiders (2008)	0 (0)	None	3, heterotopic ossification
Forthman (2007)	9 (40.9)	4, ulnar neuropathy (ulnar nerve transposition)	6, grade I arthrosis^a^
		3, stiffness (contracture release)	1, grade II arthrosis^a^
		1, instability due to noncompliance + inappropriate arm use (total elbow arthroplasty)	
		1, dislocation due to accident (interposition arthroplasty)	
Egol (2007)	5 (17.2)	1, ulnohumeral articulation resubluxation (external fixator replacement + radial head excision and replacement + elbow release)	18, heterotopic ossification
		(radial head replacement)	3, ulnar nerve neuritis
		1, painful prosthesis loosening (radial head prosthesis removal)	1, complex regional pain syndrome (stellate ganglion block)
		2, stiffness (elbow release)	
		1, NA (elbow release + radial head replacement)	
van Riet (2005)	3 (50.0)	1, severe pain and limited range of motion (total elbow arthroplasty)	1, heterotopic ossification
		1, instability (additional LCL reconstruction via semitendinosus)	1, resorption of coronoid graft + severe osteoarthritis
		1, irritated pin sites in external fixator (hardware removal + elbow release)	
Pugh (2004)	8 (22.2)	4, limited range of motion (hardware removal + elbow release)	3, heterotopic ossification
		2, radioulnar synostosis (synostosis resection + contracture release + metal radial head removal)	
		1, posterolateral rotator instability (articulated external fixator)	
		1, wound infection (surgical debridement + antibiotics)	
Ring (2002)	6 (54.5)	1, radioulnar synostosis (synostosis resection + elbow capsular release)	1, neuropathic arthropathy
		5, redislocation (4 fixation of ulnohumeral joint with pins, 1 total elbow arthroplasty)	10, ulnohumeral arthrosis^a^

LCL: lateral collateral ligament; MCL, medial collateral ligament; NA: not available.

a Based on Broberg and Morrey criteria.

The most common complications that did not require reoperation were heterotopic ossification (reported by 39 of 312 [12.5%] patients in 10 of 16 studies) and arthrosis (reported by 35 of 312 [11.2%] patients in 4 of 16 studies). In the study reported by Toros et al. [23], 6 of 16 (37.5%) patients experienced grade I arthrosis. In the study reported by Garrigues et al. [22], 5 of 40 (12.5%) patients experienced heterotropic ossification. In the study reported by Seijas et al. [18], 4 of 18 (22.2%) patients experienced heterotropic ossification. In the study reported by Lindenhovius et al. [25], 9 of 18 (50.0%) patients experienced grade I arthrosis and 3 (16.7%) experienced grade II arthrosis. In the study reported by Forthman et al. [26], 6 of 22 (27.3%) patients experienced grade I arthrosis and 1 (4.5%) experienced grade II arthrosis. In the study reported by Egol et al. [14], 18 of 37 (48.6%) patients experienced heterotropic ossification. In the study reported by Ring et al. [11], 10 of 11 (90.9%) patients experienced ulnohumeral arthrosis.

## Discussion

In this systematic review, we examined functional outcomes and complications after surgical repair of TTIE. A total of 16 studies, almost exclusively retrospective in design, involving more than 300 patients were found to be eligible for inclusion in our review. Overall, functional outcomes were satisfactory, whereas complications (including both those requiring reoperation and those not requiring reoperation) were relatively common.

We found that functional outcomes, as determined by assessing Mayo elbow performance, Broberg-Murray, and/or DASH scores were consistently satisfactory. Indeed, with regards to Mayo elbow performance and Broberg-Murray scores, approximately 70% or more of patients had good to excellent scores. Further, less than 10% of patients had poor scores. The findings of our systematic review are consistent with those of an earlier systematic review of studies published before 2009, in which the majority of Mayo elbow performance and Broberg-Murray scores were excellent or good [6]. Hence, application of current surgical strategies/technology would appear to have maintained, rather than increased, the proportion of patients experiencing positive outcomes after management of TTIE. Further refinement of surgical/management strategies in the future will hopefully decrease the proportion of patients who experience fair or poor outcomes.

Although a relatively high proportion of patients had satisfactory functional outcomes, many patients experienced complications, including ulnar neuropathy, elbow joint stiffness, heterotopic ossification, and arthrosis. Indeed, overall, slightly less than one-third of patients required reoperation due to complication(s), typically due to instability or stiffness-related problems. There was no clear chronological trend in the occurrence of complications i.e., the rate of complications did not obviously decrease with time/presumable advances in management strategies/technology. This is despite the publication of an algorithm for the surgical management of TTIE and excellent review on the topic by Mathew et al. in 2009 [2]. Interestingly, 3 studies did not report that any patients required reoperation for the management of complications [15], [16], [23]. Two of these studies [16], [23], however, included a small number (≤16) of patients. The other study, reported by Zeiders and Patel [15] involved 32 patients who were operated on following preoperative planning using three-dimensional computerized tomographic reconstruction and use of a treatment algorithm. Several patients in this study experienced heterotopic ossification; however, there was no mention of any complications requiring reoperation. This is unusual for a study involving a relatively (for this injury) large number of patients. In general, the continuing high rate of complications experienced by patients after surgical management of TTIE is concerning and signifies that further refinement of surgical approaches and preoperative and postoperative management are needed.

Our systematic review has several limitations that should be considered when interpreting the findings. Firstly, all except for 1 study [15] were retrospective in design. As such, the overall strength of evidence from these studies is not of the highest quality. Evidence from additional prospective studies would be welcome. Secondly, although our review included a relatively large number of studies (N = 16), the overall number of patients was not particularly high at just over 300. Although results from larger scale studies would provide more definitive evidence, such studies are unlikely to occur because terrible triad injuries of the elbow are not sufficiently common. Finally, the studies included in our review lacked homogeneity in a number of areas. Surgical approaches and management varied between studies, as did type of radial head and coronoid fractures, and the length of follow up. These differences may have affected both functional outcomes and complications to some extent. We must emphasize, however, that despite the lack of homogeneity, the overall results were quite compelling in that functional outcomes were consistently satisfactory for most patients and that complications were common.

In summary, the results of this systematic review indicate that most patients experience satisfactory functional outcomes following surgery for TTIE. Unfortunately, a relatively high proportion of patients continue to experience complications after surgery that often requires reoperation. Further refinements in surgical techniques and pre and postoperative management are needed to reduce complications.

## Supporting Information

Checklist S1 (DOC)
